# Mechanism of Two-/Four-Electron Reduction of Nitroaromatics by Oxygen-Insensitive Nitroreductases: The Role of a Non-Enzymatic Reduction Step

**DOI:** 10.3390/molecules23071672

**Published:** 2018-07-09

**Authors:** Benjaminas Valiauga, Lina Misevičienė, Michelle H. Rich, David F. Ackerley, Jonas Šarlauskas, Narimantas Čėnas

**Affiliations:** 1 Department of Xenobiotics Biochemistry, Institute of Biochemistry of Vilnius University, Saulėtekio 7, LT-10257 Vilnius, Lithuania; benjaminas.valiauga@bchi.vu.lt (B.V.); lina.miseviciene@bchi.vu.lt (L.M.); jonas.sarlauskas@bchi.vu.lt (J.Š.); 2 School of Biological Sciences, Victoria University of Wellington, Kelburn Parade, Wellington 6140, New Zealand; michelle.rich@vuw.ac.nz (M.H.R.); david.ackerley@vuw.ac.nz (D.F.A.)

**Keywords:** nitroreductase, aromatic nitrocompounds, reduction mechanism

## Abstract

Oxygen-insensitive NAD(P)H:nitroreductases (NR) reduce nitroaromatics (Ar-NO_2_) into hydroxylamines (Ar-NHOH) through nitroso (Ar-NO) intermediates. Ar-NO may be reduced both enzymatically and directly by reduced nicotinamide adenine dinucleotide or its phosphate NAD(P)H, however, it is unclear which process is predominant in catalysis of NRs. We found that *E. coli* NR-A (NfsA) oxidizes 2 mol of NADPH per mol of 2,4,6-trinitrotoluene (TNT) and 4 mol of NADPH per mol of tetryl. Addition of ascorbate, which reduces Ar-NO into Ar-NHOH, changes the stoichiometry NADPH/Ar-NO_2_ into 1:1 (TNT) and 2:1 (tetryl), and decreases the rate of NADPH oxidation. Ascorbate does not interfere with the oxidation of NADPH during reduction of quinones by NfsA. Our analysis of ascorbate inhibition patterns and both enzymatic and non-enzymatic reduction of nitrosobenzene suggests that direct reduction of Ar-NO by NADPH rather than enzymatic reduction is the predominant mechanism during nitroaromatic reduction.

## 1. Introduction

**Scheme 1:**
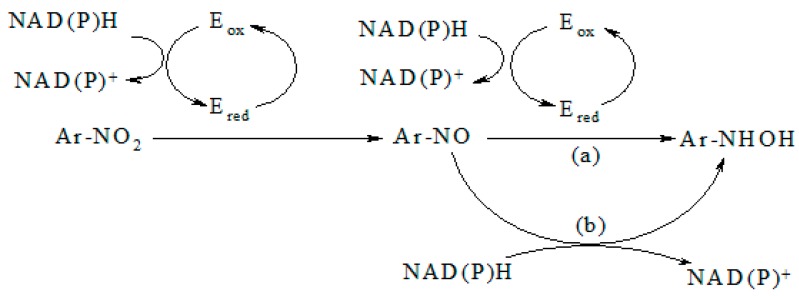
Pathways of enzymatic (a) and nonenzymatic (b) formation of Ar-NHOH from Ar-NO.

The toxic and/or therapeutic action of nitroaromatic compounds (Ar-NO_2_) is most frequently attributed to the enzymatic reduction of their nitro group(s). The single-electron reduction of nitroaromatics to their anion-radicals by flavoenzyme electrontransferases, e.g., NADPH: cytochrome P-450 reductase (P-450R) or ferredoxin: NADP^+^ reductase (FNR) initiates their redox cycling in aerobic conditions, and, subsequently, oxidative stress [1]. The two-electron reduction of nitroaromatics by bacterial nitroreductases (NRs) or mammalian DT-diaphorase results in the formation of nitroso-(Ar-NO), and, subsequently, hydroxylamine (Ar-NHOH) products (Scheme 1), which alkylate DNA and other biomolecules [2]. Apart from their role in the cytotoxicity of nitroaromatics, the latter reactions are important in the biodegradation of toxic environmental pollutants such as the polynitroaromatic explosives 2,4,6-trinitrotoluene (TNT) or 2,4,6-trinitrophenyl-*N*-methylnitramine (tetryl) [3,4].

Oxygen-insensitive NRs generally contain flavin mononucleotide FMN in the active center, and use NAD(P)H as a reducing substrate. Their reaction mechanisms and substrate specificity have been extensively studied by steady and presteady state kinetic and crystallographic methods [5,6,7,8,9]. The obligatory two-electron (hydride) reduction of nitroaromatics and quinones by NRs is attributed to an extreme instability of their FMN semiquinone redox state [10].

In spite of extensive studies of two-/four-electron reduction of nitroaromatics by NRs (Scheme 1), the pathways of formation of Ar-NHOH from Ar-NO intermediates are not well understood. Nitroso compounds possess higher redox potentials than the parent nitrocompounds [11]. Consistent with this, nitrosobenzene was suggested to be by 10^3^–10^4^ times faster than nitrobenzene in oxidation of *Enterobacter cloacae* NR or *Escherichia coli* NR-B [6,12]. For this reason, it was postulated that Ar-NO intermediates are reduced in a second faster enzyme catalytic cycle (Scheme 1, pathway (a)). However, accurate determination of the kinetic parameters of this cycle was complicated by a rapid parallel direct oxidation of NADH by nitrosobenzene [12]. Thus, it is possible that this reaction (Scheme 1, pathway (b)) plays a significant role in the formation of Ar-NHOH during the catalysis of NRs. Further evidence for this supposition is provided by reports of the direct oxidation of NAD(P)H by 1-nitroso-2-naphthol [13], and 5-(aziridin-1-yl)-2-nitro-4-nitrosobenzamide [14].

In addition to NAD(P)H, nitrosobenzenes are rapidly reduced by other reductants, including ascorbate [15]. In this work, we found that ascorbate reduced the stoichiometry and the rate of NADPH oxidation in reactions containing nitroaromatic substrates and *E. coli* NR-A (NfsA), and did not inhibit the analogous reactions of single-electron transferring flavoenzymes P-450R and FNR. Our interpretation of these inhibition patterns is that the non-enzymatic reduction of Ar-NO plays a dominant role in this step of the reduction pathway.

## 2. Results

**Figure 1:**
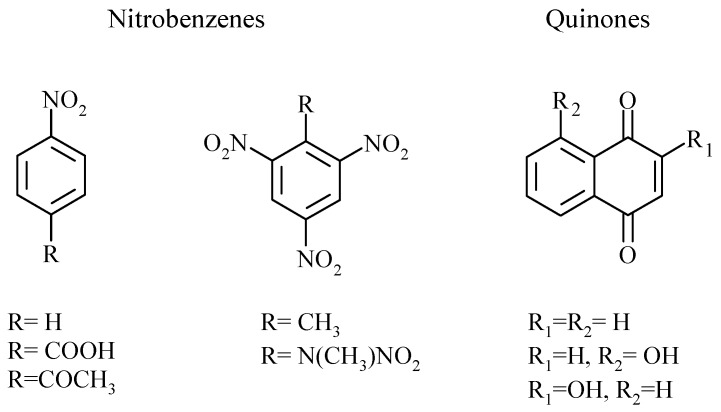
Formulae of oxidants studied in this work.

In this work, we used several nitroaromatic substrates of NfsA whose kinetic properties had been determined previously [9] (Figure 1). The NfsA-catalyzed oxidation of NADPH and subsequent reduction of these nitroaromatic substratesfollows a ping-pong mechanism. A clear trend was evident for increasing substrate reactivity (*k*_cat_/*K*_m_) correlating with increased single-electron reduction potential (*E*^1^_7_) (Table 1).

**Figure 2:**
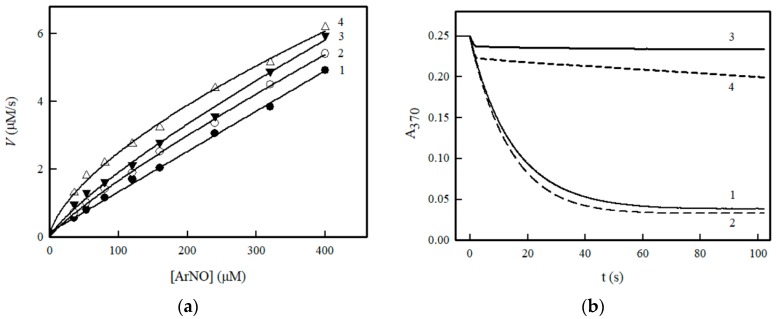
(**a**) Rates of oxidation of 100 µM NADPH by nitrosobenzene in the absence (1) and presence of NfsA (2–4). Enzyme concentration, 10 nM (2), 20 nM (3), and 40 nM (3); (**b**) Kinetics of oxidation of 100 µM NADPH by 400 µM nitrosobenzene in the absence (1,3) and presence (2,4) of 40 nM NfsA. Ascorbate (final concentration, 2 mM) was added into a syringe containing NADPH (3,4).

First, we examined the oxidation of NADPH by nitrosobenzene. As expected for a bimolecular process, the initial reaction rates at fixed NADPH were proportional to concentration of nitrosobenzene (Figure 2a). The obtained rate constant, 124.3 ± 4.5 M^−1^·s^−1^, was close to the previously established values [12,13]. The presence of NfsA accelerated the reaction (Figure 2a); however, the effect was modest at ≤40 nM enzyme concentrations. Thus, one may conclude that at NfsA concentrations typically used in steady-state assays, the rate of nonenzymatic NADPH oxidation by nitrosobenzene may even exceed the rate of enzymatic oxidation. The rate constants of NfsA-catalyzed reduction of nitrosobenzene were obtained from the differences in reaction rates in the presence and absence of enzyme (Figure 2a). We obtained values of *k*_cat(app.)_ = 55.0 ± 9.5 s^−1^ at 100 µM NADPH, and *k*_cat_/*K*_m_ = 5.7 ± 1.1 × 10^5^ M^−1^·s^−1^. The latter value is below the suggested range of *k*_cat_/*K*_m_ for the reaction of nitrosobenzene with *E. coli* NR-B, 2.2 ÷ 15.2 × 10^6^ M^−1^·s^−1^ [12]. It is likely that this reflects in part the different reactivity of NfsA and NR-B. However, it is also likely that use of the stopped-flow method (which eliminates delays in monitoring the reaction due to mixing time) has enabled a more accurate determination of the rate constants. The introduction of ascorbate into the reaction mixture rapidly suppressed the oxidation of NADPH (Figure 2b). Ascorbate would be expected to outcompete NADPH because it reduces nitrosobenzene much faster, with *k* = 2.8 × 10^3^ M^−1^·s^−1^ [15].

**Figure 3:**
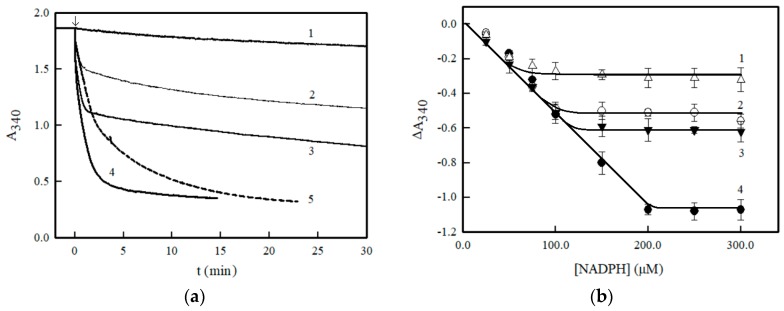
(**a**) Kinetics of oxidation of 300 µM NADPH by 50 nM NfsA in the absence of electron acceptor (1), or in the presence of 50 µM TNT (2,3), or 50 µM tetryl (4,5). The reaction mixture additionally contained 2.0 mM ascorbate in two of the reactions (2,5); (**b**) The changes in absorbance at 340 nm during the first phase of oxidation. Oxidant: 50 µM TNT (1,2), 50 µM tetryl (3,4), 2.0 mM ascorbate was present (1,3), or absent (2,4).

Because NfsA reduces nitrobenzene at a relatively slow rate, we conducted further studies of its reactions with faster oxidants possessing higher *E*^1^_7_ values (Table 1). It was found that under standard reaction conditions 2 moles of NADPH were oxidized per mole of TNT in a first reaction phase (Figure 3a), with the rate of a second phase being close to the intrinsic NADPH:oxidase activity of NfsA. This is consistent with formation of 2(4)-monohydroxyl-amino dinitrotoluene as the product of reduction of TNT by NfsA [16]. The product(s) of tetryl reduction by NfsA were not identified; however, 4 moles of NADPH were oxidized per mole of tetryl, which points to a possible formation of dihydroxylamine (Figure 3a). A similar multiphasic oxidation of excess NADH by TNT and tetryl has been observed in reactions catalyzed by *E. cloacae* NR [8]. In the absence of enzyme and the presence of an NADPH regeneration system, 50 µM TNT did not form 340 nm absorbing products, and 50 µM tetryl reduction products increased the 340 nm absorbance only marginally, by ~0.05 in 10 min. The 2:1 and 4:1 ratio of NADPH oxidized per mole of oxidant was further confirmed by the dependence of the amplitude of the first oxidation phase on NADPH concentration (Figure 3b). Importantly, the presence of ascorbate changed the NADPH/Ar-NO_2_ stoichiometry to 1:1 (TNT) and 2:1 (tetryl), and decreased the reaction rate (Figure 3a,b). In empirical pilot tests, maximal inhibition was observed at 0.5–0.7 mM ascorbate, therefore, in all subsequent experiments 2.0 mM ascorbate was used.

**Figure 4:**
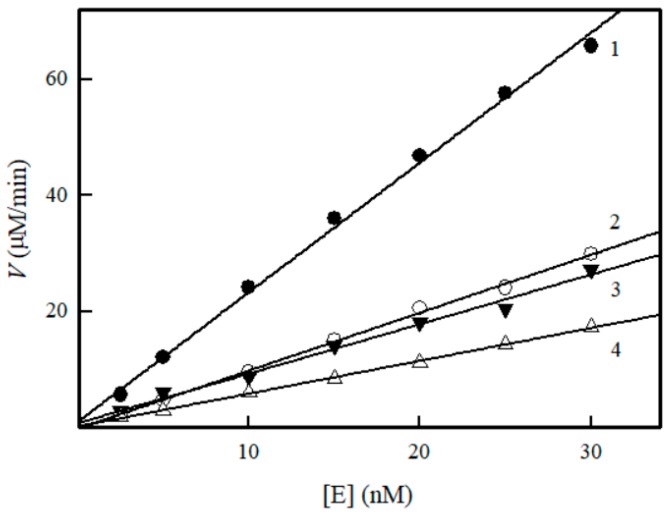
Dependence of NADPH oxidation rate on NfsA concentration in the absence (1,3) or presence (2,4) of 2.0 mM ascorbate. NADPH concentration, 200 µM, 50 µM TNT (1,2) or 2.0 mM *p*-nitrobenzoic acid (3,4) were used as oxidants.

Using different nitroaromatic oxidants, ascorbate decreased the initial reaction rate in a uniform way across a wide range of NfsA concentrations (Figure 4). The ratios of reaction rate in the absence of ascorbate and its presence were independent of the concentration of NADPH used in the experiments (15–250 µM), being equal to 1.63 ± 0.04 (nitrobenzene), 1.72 ± 0.05 (*p*-nitrobenzoic acid), 2.34 ± 0.09 (*p*-nitroacetophenone), 2.32 ± 0.03 (TNT), and 2.57 ± 0.02 (tetryl).

In control experiments assessing the stability of TNT and tetryl in the presence of ascorbate, products of TNT reduction (absorbing in the range 300–380 nm) and products of tetryl reduction (absorbing in the range 340–450 nm) were observed to appear after 20–30 min and 10–15 min delay, respectively. This indicates that during 10–15 min incubation with ascorbate the initial concentrations of TNT and tetryl were almost unchanged, evidently due to the reoxidation of their anion-radicals by oxygen [17]. When monitored using an oxygen electrode it was observed that addition of 2.0 mM ascorbate caused O_2_ uptake with a rate of 2.1 ± 0.3 µM/min (50 µM TNT), and 6.0 ± 0.7 µM/min (50 µM tetryl). The O_2_ uptake measured using other nitroaromatic oxidants was even lower (not shown).

We next examined the effects of ascorbate on the steady-state kinetic parameters, *k*_cat(app.)_ and *k*_cat_/*K*_m_, of NADPH oxidation by NfsA at fixed concentrations of oxidant. We obtained similar values of *k*_cat_/*K*_m_ for NADPH using different nitroaromatic oxidants (Table 2). Importantly, it was observed that addition of ascorbate decreased both *k*_cat_/*K*_m_ and *k*_cat(app.)_ of the reactions (Table 2).

NfsA and other NRs catalyze the two-electron reduction of quinones [9,18], which may be examined as alternative oxidants. Interestingly, when various quinones (Figure 1) were used as oxidants, the *k*_cat_/*K*_m_ measured for NADPH were consistently ~2-fold lower than those obtained using Ar-NO_2_ (Table 2). Ascorbate did not substantially affect the initial rate of NfsA-catalyzed NADPH oxidation by 1,4-NQ and 5-OH-1,4-NQ, but its presence resulted in a significant data scattering. This may be explained by the single-electron reduction of naphthoquinones by ascorbate and reoxidation of their anion-radicals by oxygen [19,20]. In the presence of 2.0 mM ascorbate and 50 µM quinone in control experiments, the O_2_ uptake rates were ≥200 µM/min (5-OH-1,4-NQ) and 70 µM/min (1,4-NQ). Therefore, for these reactions only the *k*_cat(app.)_ values at saturating NADPH concentration were determined accurately (Table 2). On the other hand, the low redox cycling rate of 2-OH-1,4-NQ, ≤0.1 µM/min, enabled us to demonstrate that in this case ascorbate decreased the *k*_cat_/*K*_m_ of NADPH only by 16% (Table 2).

In preliminary experiments, we observed that ascorbate decreased by ~2-fold the rates of TNT- and tetryl-dependent oxidation of NADPH by another two-electron transferring flavoenzyme, *E. cloacae* NR-B (data not shown). Importantly, ascorbate did not affect the rates of analogous reactions of single-electron transferring flavoenzymes P-450R and FNR, proceeding at similar time scale. In the presence of 50 nM of above enzymes, 30–50 µM TNT or tetryl oxidized excess NADPH, 200 µM, in a single phase with the rates in the range of 51–14 µM/min.

## 3. Discussion

Our data provide evidence that some steps of two-/four-electron reduction of nitroaromatics by nitroreductases are largely enzyme-independent, namely the pathway of formation of Ar-NHOH from Ar-NO. In support of this, we demonstrated that under certain conditions, the rate of non-enzymatic NADPH oxidation by nitrosobenzene may exceed the rate of its enzymatic oxidation (Figure 2a). For other nitroaromatic compounds, this evidence was obtained indirectly, by the analysis of ascorbate inhibition patterns (Figure 3 and Figure 4, Table 2).

First, in NfsA-catalyzed NADPH oxidation by nitroaromatics, ascorbate decreases the amount of NADPH oxidized per Ar-NO_2_ and the reaction rate by ~2-fold (Figure 3 and Figure 4). On the other hand, it did not affect the rate of quinone-dependent NADPH oxidation by NfsA (Table 2). Thus, the effect of ascorbate on the NADPH/Ar-NO_2_ stoichiometry is consistent with the rapid reduction of Ar-NO intermediates by ascorbate [15] which withdraws them from further oxidation of NADPH. One may expect that the parallelism between the rates of NAD(P)H and ascorbate oxidation will be maintained for the nitroso derivatives of other nitroaromatics (Table 1). This is because the reactivity of nitrosobenzenes towards ascorbate and synthetic NADH derivative increases with an increase in their electron-accepting properties, and follows Hammett-type relationships with the value of ρ close to 1.0 [15,21]. On the other hand, as the nitroanion radical products of reduction generated by P-450R or FNR would be expected to be rapidly back oxidized by oxygen, the nitroso products would not be achieved, and hence ascorbate would not be expected to influence the reaction.

Second, the *k*_cat(app)_/*K*_m_ values measured for NADPH oxidation using nitroaromatic compounds as oxidants were ~2-fold higher than those measured in quinone reductase reactions (Table 2), with this difference being abrogated by ascorbate. Such a difference is inconsistent with the ping-pong reaction mechanism, where the reductive and oxidative half-reactions proceed independently, and the *k*_cat_/*K*_m_ value for the reducing substrate should not depend on the nature of the oxidant [22]. A possible occurrence of a second faster catalytic cycle, the reduction of Ar-NO by repeatedly reduced enzyme (Scheme) should not change the *k*_cat_/*K*_m_ for NADPH, because in both cycles NADPH reacts with the same enzyme form. Because nitroaromatics and other aromatic compounds bind at the nicotinamide binding pocket close to the isoalloxazine ring of FMN [7,12], it is unlikely that Ar-NO can participate in a ternary complex formation with NR. Ascorbate should also not affect the *k*_cat_ of NfsA, because this is limited by the slowest catalysis step, oxidation of the reduced enzyme by oxidants [9]. Taken together, these data argue against significant involvement of NfsA in the reduction of nitroso derivatives of examined compounds (Table 1), and for the major role of its direct reduction by NADPH (Scheme 1, pathway (b)). According to this scheme, Ar-NO should simply amplify the rate and stoichiometry of enzymatic NADPH oxidation by ~2-fold. The quantitative analysis of competition between NAD(P)H and NR for the reduction of nitroso compounds other than nitrosobenzene is complicated by the absence of relevant kinetic data. However, our findings show that the inhibition by ascorbate takes place at a wide range of NfsA concentrations (Figure 3).

Apart from clarifying the reaction mechanism, our data may be helpful in the modification of biodegradation pathways of polynitroaromatic compounds. Although nitrosobenzene is not transiently accumulated during the reduction of nitrobenzene by NR in the presence of an NADH regeneration system [6], aerobic incubation of TNT with *Pseudomonas* sp. leads to close to 25% yield of azoxyarenes, the condensation products of nitroso- and hydroxylamine metabolites [23]. Nitroso metabolites of dinitrotoluene were transiently formed by *Salmonella typhimurium* with a close to 50% yield even under anaerobic conditions [24]. In addition to reoxidation of Ar-NHOH by O_2_, a possible cause is limited NAD(P)H regeneration. In our opinion, the addition of ascorbate could significantly reduce the transient accumulation of the above groups of metabolites.

## 4. Materials and Methods

### 4.1. Enzymes and Chemicals

*E. coli* NfsA was purified as described [25]. Recombinant human NADPH:cytochrome P-450 reductase (P-450R) was a generous gift of Dr. Aleksey Yantsevich (Institute of Bioorganic Chemistry, Minsk, Belarus), FNR from *Plasmodium falciparum* was a generous gift of Dr. Alessandro Aliverti (Universita degli Studi di Milano, Milano, Italy), and “retro” NR-B from *E. cloacae* was a generous gift of Dr. Ronald L. Koder (The City College of New York, New York, NY, USA). The enzyme concentrations were determined spectrophotometrically using ε_460_ = 12.5 mM^−1^·cm^−1^ (NfsA), ε_460_ = 22 mM^−1^·cm^−1^ (P-450R), ε_461_ = 10.1 mM^−1^·cm^−1^ (FNR), and ε_454_ = 14.3 mM^−1^·cm^−1^ (NR-B). TNT, tetryl and nitrosobenzene were synthesized according to [26,27,28], respectively. All other compounds were obtained from Sigma-Aldrich (St. Louis, MO, USA) and used as received.

### 4.2. Enzymatic Assays

Steady-state kinetic measurements were performed spectrophotometrically in 0.1 M K-phosphate (pH 7.0), containing 1 mM EDTA, at 25 °C, using a PerkinElmer Lambda 25 spectrophotometer (PerkinElmer, Waltham, MA, USA). The stock solutions of oxidants were prepared in DMSO (dilution factor 100). The initial rates of enzymatic NADPH-dependent nitroreduction were determined by monitoring the oxidation of NADPH (∆ε_340_ = 6.2 mM^−1^ cm^−1^). Data were corrected for the intrinsic NADPH:oxidase activity of the enzymes, measured in oxidant-free control reactions. The steady-state parameters of reactions, the turnover numbers (*k*_cat(app.)_) and the bimolecular rate constants (or catalytic efficiency constants, *k*_cat_/*K*_m(app.)_) of NADPH at fixed concentration of the oxidants correspond to the reciprocal intercepts and slopes of Lineweaver-Burk plots, [E]/*v* vs. 1/[NADPH], where *v* is the reaction rate, and [E] is the enzyme concentration. *k*_cat(app.)_ represents the number of molecules of NADPH oxidized by a single active center of the enzyme per second. The kinetic parameters were obtained by the fitting of kinetic data to the parabolic expression using SigmaPlot 2000 (version 11.0, Systat Software, San Jose, CA, USA). In some experiments, as noted in the main text, an NADPH regeneration system (20 µM NADPH, 10 mM glucose-6-phosphate, and 0.5 mg/mL glucose-6-phosphate dehydrogenase) was used. Because nitrosobenzene possesses significant absorbance at 340 nm, the initial rates (0–5 s) of oxidation of NADPH by nitrosobenzene were determined according to ∆ε_370_ = 2.5 mM^−1^∙cm^−1^ using a stopped-flow SX.17 MV spectrophotometer (Applied Photophysics, Leatherhead, UK). When necessary, NfsA was added to a syringe containing nitrosobenzene, and ascorbate was added to a syringe containing NADPH. Oxygen consumption during the reactions of TNT, tetryl and naphthoquinones with ascorbate was monitored using a Digital Model 10 Clark electrode (Rank Brothers Ltd., Bottisham, UK). The initial O_2_ concentration at 25 °C was assumed to be 250 µM.

## Figures and Tables

**Table 1:** The single-electron reduction potentials (*E*^1^_7_) of nitroaromatic compounds and the steady-state rate constants of their reduction by NfsA [9].

No	Oxidant	*E*^1^_7_ (V)	*k*_cat_ (s^−1^)	*k*_cat_/*K*_m_ (M^−1^·s^−1^)
1	Tetryl	−0.16	110.0 ± 13.0 ^1^	7.9 ± 0.9 × 10^6^
2	TNT	−0.25	135.1 ± 12.3 ^1^	2.7 ± 0.3 × 10^6^
3	*p*-Nitroacetophenone	−0.36	59.4 ± 5.2 ^2^	1.5 ± 0.2 × 10^5^
4	*p*-Nitrobenzoic acid	−0.43	64.3 ± 7.0 ^2^	2.5 ± 0.5 × 10^3^
5	Nitrobenzene	−0.49	14.2 ± 2.0 ^2^	1.5 ± 0.3 × 10^3^

^1^ Determined at infinite NADPH concentration; ^2^ *k*_cat(app.)_, determined at [NADPH] = 100 µM.

**Table 2:** The steady-state rate constants of NADPH oxidation by NfsA using various oxidants in the absence and presence of 2.0 mM ascorbate.

No	Oxidant	−Ascorbate		+Ascorbate	
		*k*_cat(app.)_ (s^−1^)	*k*_cat_/*K*_m_ (M^−1^·s^−1^)	*k*_cat(app.)_ (s^−1^)	*k*_cat_/*K*_m_ (M^−1^·s^−1^)
1	TNT (50 µM)	52.0 ± 5.5	3.85 ± 0.36 × 10^6^	23.0 ± 3.5	1.56 ± 0.22 × 10^6^
2	Tetryl (50 µM)	41.8 ± 7.0	3.78 ± 0.50 × 10^6^	16.1 ± 3.0	2.08 ± 0.23 × 10^6^
3	*p*-Nitroacetophenone (0.5 mM)	50.8 ± 6.2	3.20 ± 0.42 × 10^6^	20.1 ± 3.5	1.62 ± 0.21 × 10^6^
4	*p*-Nitrobenzoic acid (2.0 mM)	30.6 ± 4.5	3.30 ± 0.42 × 10^6^	19.7 ± 1.2	2.05 ± 0.30 × 10^6^
5	Nitrobenzene (1.5 mM)	4.6 ± 0.3 ^1^	n.d. ^2^	3.1 ± 0.2 ^1^	n.d. ^2^
6	5-OH-1,4-NQ (50 µM	19.4 ± 2.9	1.60 ± 0.22 × 10^6^	18.3 ± 2.2 ^1^	n.d. ^3^
7	1,4-NQ (50 µM)	26.0 ± 3.2	1.52 ± 0.18 × 10^6^	22.0 ± 3.0 ^1^	n.d. ^3^
8	2-OH-1,4-NQ (20 µM)	26.5 ± 1.4	1.70 ± 0.22 × 10^6^	26.2 ± 3.7	1.46 ± 0.23 × 10^6^

^1^ The rate at saturating NADPH concentration, 250 µM; ^2^ Not determined due to low *K*_m_ values for NADPH; ^3^ Not determined due to data scattering.

## Abbreviations

The following abbreviations are used in this manuscript:

1,4-NQ	1,4-naphthoquinone
2-OH-1,4-NQ	2-hydroxy-1,4-naphthoquinone
5-OH-1,4-NQ	5-hydroxy-1,4-naphthoquinone;
Ar-NO_2_	aromatic nitrocompound
FMN	flavin mononucleotide
FNR	ferredoxin:NADP^+^ reductase
*k*_cat(app.)_	turnover number
*k*_cat_/*K*_m(app.)_	bimolecular rate constant
NAD(P)H	reduced nicotinamide adenine dinucleotide or its phosphate
NfsA	*E. coli* nitroreductase-A
P-450R	NADPH:cytochrome P-450 reductase
TNT	2,4,6-trinitrotoluene
